# Cell Sources for Human In vitro Bone Models

**DOI:** 10.1007/s11914-020-00648-6

**Published:** 2021-01-15

**Authors:** Sana Ansari, Keita Ito, Sandra Hofmann

**Affiliations:** Orthopaedic Biomechanics, Department of Biomedical Engineering and Institute for Complex Molecular Systems, Eindhoven University of Technology, P.O. Box 513, 5600 MB Eindhoven, the Netherlands

**Keywords:** In vitro bone model, Bone marrow, Peripheral blood, Mesenchymal stem cell, Hematopoietic stem cell, Personalized medicine

## Abstract

**Purpose of Review:**

One aim in bone tissue engineering is to develop human cell-based, 3D in vitro bone models to study bone physiology and pathology. Due to the heterogeneity of cells among patients, patient’s own cells are needed to be obtained, ideally, from one single cell source. This review attempts to identify the appropriate cell sources for development of such models.

**Recent Findings:**

Bone marrow and peripheral blood are considered as suitable sources for extraction of osteoblast/osteocyte and osteoclast progenitor cells. Recent studies on these cell sources have shown no significant differences between isolated progenitor cells. However, various parameters such as medium composition affect the cell’s proliferation and differentiation potential which could make the peripheral blood-derived stem cells superior to the ones from bone marrow.

**Summary:**

Peripheral blood can be considered a suitable source for osteoblast/osteocyte and osteoclast progenitor cells, being less invasive for the patient. However, more investigations are needed focusing on extraction and differentiation of both cell types from the same donor sample of peripheral blood.

## Introduction

Bone is a complex multifunctional organ that sustains the integrity of the vertebrate skeleton, provides mechanical support for locomotion, protects internal organs, and acts as a mineral storage [1]. Throughout life, bone tissue continuously undergoes a physiological process called bone remodeling to adapt to environmental changes, repair old and damaged bone, and maintain its shape and strength. Bone remodeling occurs via balanced activities of its specialized cells which are tightly regulated and controlled through biochemical pathways [2]. In vivo*,* bone remodeling is composed of four consecutive phases: recruitment and activation of mononuclear progenitor cells, resorption of the organic and inorganic matrix of bone by mature osteoclasts, preparation of the resorbed surface of matrix deposition, and deposition of new bone by osteoblasts [1, 3].

### Bone Cells

#### Osteoblasts

**Fig. 1:**
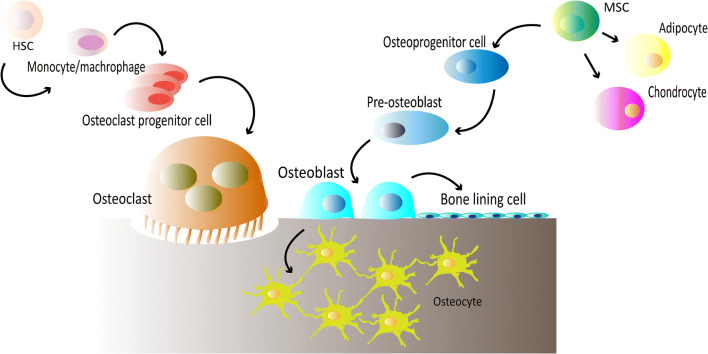
Bone progenitor cells and their differentiation into osteoblasts/osteocytes and osteoclasts

Osteoblasts are bone-forming cells derived from mesenchymal stem cells (MSCs). MSCs differentiate towards osteoblasts under appropriate mechanical and/or biochemical stimuli [4, 5]. Osteoblasts are responsible to produce the organic matrix of bone extracellular matrix composed of mainly collagen type I and a small percentage of non-collagenous proteins (NCPs) [6]. Moreover, they are involved in inorganic matrix deposition through mechanisms in which NCPs play important roles [7, 8]. At the end of the bone-forming phase, osteoblasts can have one of the following fates: become embedded in the mineralized matrix and differentiate towards osteocytes, transform into inactive bone-lining cells, or undergo apoptosis (Fig. 1) [9].

#### Osteocytes

Osteocytes as terminally differentiated osteoblasts form 95% of the cellular component of bone; thus, they are the most abundant bone cell type [10]. During bone formation, a large portion of osteoblasts becomes embedded in the mineralized matrix, decrease their cell body volume, and attain a stellar shape morphology with long processes which form a network with their neighboring cells and cells on the bone surface [10]. These cells are thought to orchestrate the activities of bone formation and resorption by translating mechanical loading into biochemical signals [11, 12].

#### Osteoclasts

Osteoclasts are bone-resorbing cells that dissolve the inorganic matrix and enzymatically degrade extracellular matrix proteins by secreting acid and lytic enzymes [13]. These cells are large, multinucleated cells originating from the monocyte/macrophage lineage which differentiate from hematopoietic stem cells (HSCs) (Fig. 1). These stem cells are situated in bone marrow and can be mobilized into the peripheral blood [14]. Osteoclast differentiation and activation are thought to be regulated by neighboring stromal cells and osteoblasts [15].

#### Others

Besides the cell types that are involved in bone remodeling process, bone consists of other cell types which are less known to have a direct role in the bone remodeling process; they will not be addressed in this review. These cells are for example bone-lining cells, which are inactive osteoblasts at the end of the bone formation phase, chondrocytes, and endothelial and perivascular cells due to the vascularized nature of bone tissue [10, 16, 17].

### Bone Metabolic Diseases

Disturbing the bone remodeling process results in the development of metabolic bone diseases including osteoporosis characterized by an altered bone turnover balance as a result of high osteoclast activity and impaired bone formation. Osteoporosis is the most common bone metabolic disease. It is characterized by decreased bone strength and increased bone fracture risk [18, 19]. Apart from osteoporosis, there are more diseases related to an impaired bone remodeling process including osteopetrosis, Paget’s disease, renal osteodystrophy, and rickets. They are less prevalent, which limits our current knowledge on their pathology and their efficient treatment [20]. Thus, the development of in vitro models that mimic bone-related pathologies could enhance the understanding of these diseases and the design of more efficient treatments.

### The Need for Personalized In Vitro Models

The current gold standard in developing novel treatments for bone pathologies and pre-clinical drug screening is using animal models. However, these often fail to represent human conditions due to interspecies differences in physiology [21, 22]. Moreover, the need for indicating the appropriate species to model a specific disease, ethical concerns due to genetic mutations and/or nutrient deficiency to induce the disease and high costs of maintenance limit the use of animals as models and thus our knowledge on specific bone metabolic diseases. Animal models often result in poor translation of pre-clinical studies to human clinical trials and promising new treatments might fail prior to clinical testing [22–24]. The development of new therapies requires an in-depth and detailed understanding of bone physiology and pathology and how the different cells are affected in their interaction. Over the past few years, bone tissue engineering techniques have been applied to create 3D in vitro bone models based on human cells that can be used as an alternative to in vivo models [18, 23]. These in vitro bone models require the (co-)culture of the specific bone cells to work closely together under physiological conditions. Because there is a large cell heterogeneity among patients and their diverse characteristics caused by diseases include changes in cell receptors, there is a need to use patient-specific cells for personalized in vitro bone models [19, 25]. Thus, in order to represent the patient’s bone biological system, representative in vitro models require the patient’s own cells. The ideal and efficient way to achieve this approach is to isolate progenitor cells of osteoblasts and osteoclasts with high efficiency, to expand them in vitro, and to differentiate them towards osteoblasts/osteocytes and osteoclasts, respectively, ideally from one cell source with minimal invasiveness for the patient (i.e., either from peripheral blood or from bone marrow).

This review attempts the following: to (a) briefly identify what kind of cells can be used for bone-related studies, (b) explain the importance of progenitor cells as the most promising cell types for developing in vitro bone models, (c) discuss bone marrow and peripheral blood as sources to obtain both osteoblasts/osteocytes and osteoclasts progenitor cells, and (d) finally, the isolation method, proliferation capacity, and differentiation potential of progenitor cells from bone marrow and peripheral blood are discussed.

## Cells in Development of In Vitro Bone Models

Advancement in development of in vitro bone models requires the selection of suitable cell models which can behave similarly to the ones in vivo*.* Cells that have been used in bone-related studies might be originated from one of the following: immortalized cell lines, primary cells which are isolated directly from the tissue, induced pluripotent stem cells (iPSCs), and progenitor cells.

Immortalized cell lines such as MC3T3-E1, MLO-A5, and MG-63 have been used extensively in bone tissue engineering due to their ease of access, high expansion capacity, and reproducibility of outcomes [3]. However, these cell models do not always behave similarly to primary bone cells [26]. For instance, in murine calvarial cell line MC3T3-E1, the gene expression of specific transcripts coding for extracellular matrix proteins such as osteopontin may differ compared with primary osteoblastic cells [27, 28]. Besides, as immortalized cell lines are not patient-specific, it is clear that they cannot be considered as suitable candidates for personalized human in vitro bone models.

iPSCs, which are generated by transferring a mixture of nuclear transcriptional factors including Oct4, Sox2, Klf4, and c-Myc to human primary cells, exhibit high similarity to human embryonic stem cells [29]. Due to their robust proliferation capacity, differentiation potential into many cell types, and the ability to generate patient-specific stem cells, iPSCs gained high interest in disease modeling, drug screening, and transplantation therapies [29]. Several studies have shown the ability of iPSCs to differentiate into osteoblasts and osteoclasts, suggesting that iPSCs could be considered as a cell model for the generation of in vitro bone models [30–33]. However, approaches to generate iPSCs might be complex, expensive, and time-consuming with low reprogramming efficiency and possible alternations of gene expression profiles and pathways, which make iPSCs less appropriate for development human in vitro bone models, at least for the moment.

Primary osteoblasts and osteocytes can both be directly isolated from bone tissue and provide an alternative to cell lines for bone-related studies. Several protocols and methods are available for the isolation of human osteoblasts including enzymatic digestion and spontaneous outgrowth cultures from bone biopsies [34, 35]. Isolation of primary osteocytes is more challenging due to their location within the mineralized bone matrix which requires multiple digestion and decalcification steps [36]. As an alternative, human osteocytes can be obtained in culture through differentiation of isolated osteoblasts under osteogenic stimulation [37, 38•]. For primary osteoclasts, it has been reported in early studies that they can be isolated from human bone tissue [39, 40]. However, isolation of primary osteoclasts from bone tissue requires multiple steps which might affect the number of extracted cells and their survival rate [41].

Primary cells have greatly enhanced the knowledge of bone biology; for instance, a recent study has shown development of an in vitro model to investigate the interaction of primary human osteoblasts and osteocytes [38•]. But due to their need for a bone biopsy, slow proliferation rate, short life-span, decreased doubling time after two or three passages, long isolation procedures, limited accessibility, restricted pool of potential donors (they are usually acquired during orthopedic surgery) [34, 42], their use for developing personalized human in vitro models is restricted.

The use of progenitor cells of the bone-specific cell types could be more promising to develop human in vitro bone models. MSCs are osteoblast/osteocyte progenitor cells which were primarily extracted from bone marrow and later from other tissues such as adipose tissue, muscle, peripheral blood, dental pulp as adult tissue sources and umbilical cord, umbilical cord blood, placenta, amniotic fluid as fetal and perinatal tissue sources [43–45]. MSCs can differentiate into various lineages such as adipogenic, chondrogenic, and osteogenic lineage under appropriate stimuli [46–48]. In addition to their multi-potency, their availability and relative ease of isolation and expansion have made them popular for use in many in vitro models. Bone marrow-derived MSCs have shown significant roles in bone regeneration and fracture repair in vivo; furthermore, in vitro studies demonstrated a high osteogenic differentiation capacity under biochemical and/or mechanical stimuli [49–53]. In bone tissue engineering, bone marrow has so far probably gained the greatest attention as a source of MSCs, but due to the invasive and painful procedure of bone marrow aspirate collection which can also cause donor site morbidity, other adult and fetal tissue sources have been studied as the source of MSCs [54]. For instance, several studies have indicated the osteogenic differentiation and bone formation potential of adipose-derived MSCs, which can be isolated from the tissue obtained during liposuction, lipoplasty, or lipectomy procedures with less discomfort and complications compared with bone marrow aspirate collection [55, 56]. Further, MSCs derived from umbilical cord blood and peripheral blood with less invasive cell collection methods have also shown their potential for bone defect repair [57–59].

Hematopoietic stem cells (HSCs) are multi-potent and self-renewing cells that can give rise to immune and blood cells [60, 61]. HSCs are primarily located in the bone marrow and can be mobilized into the bloodstream which makes bone marrow and peripheral blood the common tissue sources for HSC extraction [62, 63••, 64]. Moreover, it has been shown that these cells can also be isolated from umbilical cord blood [65, 66]. HSCs differentiate into the monocyte/macrophage lineage and further into osteoclasts under stimulation with receptor activator of nuclear factor kappa-B ligand (RANKL) and monocyte-colony-stimulating factors (M-CSF), both of which are secreted in vivo by osteoblasts and osteocytes [67, 68].

**Fig. 2:**
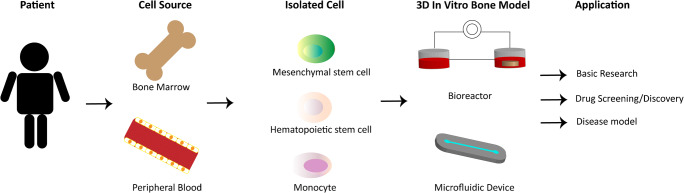
Cell sources for personalized in vitro bone models

Taken together, the most promising cell models for generation of personalized human in vitro bone models are progenitor cells. To develop these models, the patient’s own progenitor cells should ideally be extracted from one source which makes the procedure more convenient for the patient, as well as results in less demanding clinical procedure. Among all adult tissue sources, due to the possibility to extract both MSCs and HSCs from bone marrow and peripheral blood, they can be considered being the most suitable sources for the isolation of osteoblast/osteocyte and osteoclast progenitor cells (Fig. 2).

## Bone Marrow–Derived MSCs vs. Peripheral Blood–Derived MSCs

The frequency of MSCs derived from bone marrow and peripheral blood is very low, representing approximately 0.001–0.01% and 0.000001% of isolated mononuclear cells, respectively [69, 70]. The number of isolated MSCs can be changed depending on the gender, donor age, health condition, and in case of bone marrow-derived MSCs, skeletal site of isolation such as anterior or posterior iliac crest, vertebral body or femoral head [71–73]. It has been shown that the frequency of circulating MSCs in peripheral blood can be enhanced in response to pathological conditions such as bone fracture, osteoporosis, breast cancer, and bone sarcomas; for instance, a 9-fold increase in the number of MSCs has been reported in the bloodstream of patients with osteosarcoma compared with control subjects [74–78]. This could be as a result of released cytokines and chemical signals to recruit MSCs and mobilize them into the bloodstream. Several methods have been used to mimic these signals to increase the number of MSCs in blood circulation such as administration of granulocyte-colony-stimulating factor (G-CSF) and activation of the sympathetic nervous system by electro-acupuncture [79–82]. These methods could result in elevated number of isolated peripheral blood-derived MSCs which might be an advantage to develop patient-specific in vitro bone models; however, due to the possible side effects of stimulating the migration of cells from bone marrow to peripheral blood, it might not be ethical for the donors and also not applicable for patients with specific diseases [83]. To isolate MSCs from bone marrow and peripheral blood, several protocols have been used such as direct plating based on the adherence property of MSCs to the plastic surface [45, 70], density gradient centrifugation, or hemolysis to separate mononuclear cells and remove erythrocytes prior to seeding cells on a plastic surface [63, 84–88, 89•, 90, 91], using fibrin microbeads and fluorescence-activated cell sorting (FACS) to increase the purity of extracted MSCs [79, 92]. Due to the low frequency of isolated MSCs, their applicability relies on their high in vitro proliferation capacity.

The proliferation capacity of MSCs can be evaluated by calculating population doubling time [93]. Various studies on MSCs derived from bone marrow and peripheral blood have shown different doubling times for MSCs; for instance, 80 and 27 h of doubling times have been reported for bone marrow-derived MSCs and peripheral blood-derived MSCs, respectively [44]. These differences could also be as a result of donor-to-donor variability, factors such as age and health condition of the donor, passage number of cells in vitro and the use of different protocols for cell isolation and culture [76, 87, 94–101]. For instance, isolation of peripheral blood-derived MSCs based on positive expression of CD133 led to obtain MSCs with high proliferative potential in comparison with the peripheral blood-derived MSCs based on their adherence capability to plastic surface [101]. This could be due to the heterogeneous population of cells in the plastic adherence method that might interfere with proliferation capacity. While an investigation on bone marrow- and peripheral blood-derived MSCs obtained from same patients with the same isolation method and culture condition has reported no significant differences in their characteristics such as population doubling time [76], in another study, the quantity of obtained MSCs from bone marrow after two passages was 2 times higher than MSCs from peripheral blood [89•]. These differences not only could be a result of donor variation, but also of the culture condition and most importantly the medium composition. Even though these observations could suggest that the use of peripheral blood-derived MSCs in tissue engineering applications might be equally valuable as bone marrow-derived MSCs, the chosen culture conditions need to be evaluated carefully.

The in vitro osteogenic differentiation of MSCs makes them highly interesting for the development of in vitro bone models [63, 76, 89•, 102–106]. The general trend shows a beneficial osteogenic differentiation potential for bone marrow-derived MSCs based on significantly increased expression of osteoblastic specific genes, such as alkaline phosphatase (ALP) and calcium deposition compared with other tissue sources [106, 107]. On the other hand, studies on MSCs extracted from bone marrow and peripheral blood of the same patients have shown no significant differences in quantitative measurements of ALP expression and calcium content; moreover, MSCs from both sources have demonstrated positive staining for calcium deposits [76, 89]. Besides the differentiation potential of MSCs towards the osteogenic lineage, their ability to promote bone formation after in vivo implantation has been shown in various studies. The majority of these studies has been conducted using bone marrow-derived MSCs in human and animal models [108–112]. In a small number of studies, also peripheral blood-derived MSCs have been used and shown to enhance bone regeneration in critical-sized bone defects in animal models [59, 113, 114]. Taken together, peripheral blood-derived MSCs seem to exhibit similar characteristics as bone marrow-derived MSCs and can be used to develop patient-specific in vitro bone models.

The main challenge in developing in vitro bone models to represent bone remodeling is the formation of osteocytes embedded in the mineralized matrix. In vitro, human osteocytes have been obtained through the differentiation of primary osteoblasts, but so far, full differentiation of MSCs towards functional osteocytes has not been reported (Table 1). Further investigations will be needed to induce the formation of osteocytes that are embedded in their own matrix in vitro. This might be acquired for example through exposing cells to mechanical stimuli which are known to be involved in bone homeostasis and bone remodeling [3, 123].

**Table 1:** Current approaches to differentiate osteocytes in vitro

Cell	Osteoblast source	Culture substrate	Outcome	Reference
Human primary osteoblasts	Purchased from LONZA	Biphasic calcium phosphate particles	Expression of CX43, DMP1, E11, MEPE, SOST, PHEX Embedded osteocyte-like cells in collagenous matrix	[115]
Human primary osteoblasts	Femoral trabecular bone tissue from the knee region	Mineralized collagen matrix	Expression of DMP1 and FGF23 Formation of lacunae around the cell	[116]
Human primary pre-osteoblasts and mature osteoblasts	Spongious bone fragment of human femoral head	Collagen gel	Expression of E11, osteocalcin, PHEX, MEPE, RANKL Acquire stellar shape of osteocyte	[38•]
		Mineralized collagen gel	Expression of ALP, PDPN, PHEX Acquire stellar shape of osteocyte	[37]
Human primary osteoblasts	Intertrochanteric bone	2D on tissue culture plastic	Expression of E11, DMP1, SOST, OOCN, BSP1, PHEX	[117]
Human primary osteoblasts	Knee cortical	3D microfluidic perfusion device	Expression of SOST and FGF23 Form 3D cellular network Inhibit cell proliferation	[118]
Mouse primary osteoblasts	Long bone	3D microfluidic perfusion device	Expression SOST, FGF23 Form 3D cellular network Inhibit cell proliferation	[119]
Mouse primary osteoblasts	Calvarial tissue	2D culture on poly-L-lysine-coated 2-well chamber slide	Expression of ALP, DMP1, sclerostin Formation of mineralized nodules Acquire stellar shape of osteocyte	[120]
Rat primary periosteal cells	Femur bone	Fibrin hydrogel with calcium phosphate ceramic anchors	Deposition of ordered matrix containing collagen and hydroxyapatite Expression of sclerostin and PDPN Embedded cell with osteocyte morphology in the mineralized matrix	[121]
Mouse mesenchymal stem cell	Bone marrow	2D culture on tissue culture plastic	Formation of mineralized nodules Expression of E11, DMP1, PHEX, SOST, FGF23, RANKL, OPG. Acquire stellar shape of osteocyte	[122]

## Bone Marrow-Derived HSCs Vs Peripheral Blood-Derived HSCs

HSCs represent a rare population of cells in bone marrow and peripheral blood, representing less than 0.01% and less than 0.000001% of total nucleated cells, respectively [124–127]. However, the population of cells could be influenced by the age and health condition of patients and the method of cell isolation [128–131]. HSCs are primarily located in the bone marrow, but, just like MSCs, they display dynamic behavior by moving out of the bone marrow and entering into the general circulation [68, 132]. The mobilization process could be enhanced by administration of various factors and depending on the type of used pharmacological agent such as G-CSF and CXCR4 receptor antagonist AMD3100, the frequency of HSCs in peripheral blood could be elevated up to 100 times [68, 132–135]. However, the possible side effects of exposing donors to these pharmacological agents might not be ethical for the donors [83]. Luckily, monocytes that are derived from HSCs and comprise 10–20% of peripheral blood mononuclear cells can be directly isolated from peripheral blood and have been used as osteoclast precursor cells in in vitro studies [136–142]. To isolate HSCs from bone marrow and peripheral blood, several protocols have been developed including direct plating of bone marrow aspirates and blood samples on plastic surface and collecting the non-adherent cells as it has been shown that HSCs are less likely to attach to the plastic substrate compared with MSCs. The molecular and biochemical analyses on the non-adherent cells of bone marrow and peripheral blood mononuclear cells revealed that they are positive for HSC markers such as SLAM F1 [63••]. Culturing non-adherent cells in osteoclastogenesis promoting medium resulted in the differentiation of functional osteoclasts which was associated with expression of the tartrate-resistant acid phosphatase (TRAP) gene and an increased TRAP enzyme activity [62, 63, 143••, 144]. Another method is culturing mononuclear cells separated via density gradient centrifugation under osteoclastogenic culture condition which led to the generation of osteoclasts in culture [145, 146]. Moreover, HSCs and monocytes can be isolated and purified based on the expression of their own specific surface marker such as CD34 and CD14 using techniques including an automated magnetic purification system and FACS [124, 125, 136–138, 147–150].

Unlike MSCs which exhibit a high proliferation capacity and can be expanded in vitro to obtain a high number of cells for in vitro studies, the proliferation of HSCs and monocytes remains challenging. For the generation of in vitro bone models and osteoclast-related studies, freshly isolated osteoclast progenitor cells have been used in most studies [137, 151–153]. The time-consuming procedure of cell isolation might cause difficulties in obtaining a high enough number of cells; as a result, large volume of bone marrow aspirates or peripheral blood would be required. Attempts to increase the number of osteoclast precursor cells in vitro resulted in the development and use of several components and factors in culture [126]. It has been shown that combinations of growth factors and cytokines such as interleukin 6 (IL-6), interleukin 3 (IL-3), thrombopoietin (TPO), and stem cell factor (SCF) with additional molecules such as Prostaglandin E2 (PGE2), Stemregenin 1 (SR1), and UM171 could support the proliferation of HSCs in vitro [126, 154–156]*.* Monocytes have also showed an increased proliferation potential in vitro in response to macrophage-colony-stimulating factor (M-CSF), 1α,25-dihydroxyvitamin D3, and lymphokines [157–161]. However, the influence of these components on the subsequent osteoclastogenesis potential needs further investigation. Cryopreservation of osteoclast progenitor cells seems feasible as it has not affected monocyte viability and function in response to various factors [162–165]. However, further exploration is required on the osteoclast differentiation ability of cryopreserved osteoclast progenitor cells.

**Fig. 3:**
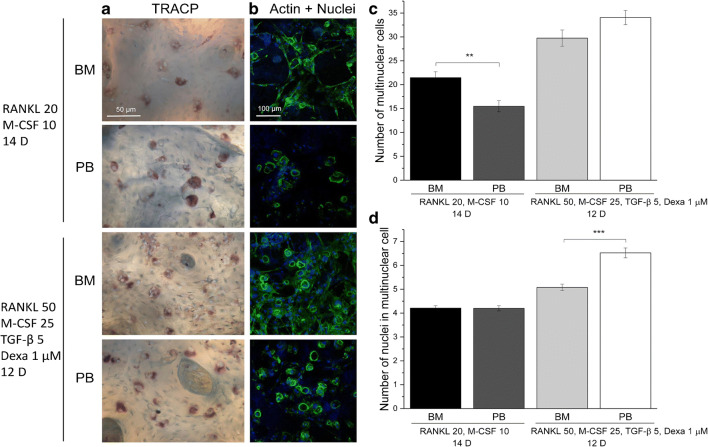
Osteoclast differentiated from bone marrow and peripheral blood cultures under different combination of growth factors. The multinuclear TRACP-positive cells are shown in **a** and the actin rings are illustrated in **b**. Higher number of multinuclear cells in bone marrow-derived cultures were obtained in the presence of RANKL and M-CSF (**c**). The number of nuclei in multinuclear cell was similar in bone marrow and peripheral blood-derived osteoclasts when only RANKL and M-CSF were used, but in the presence of dexamethasone, the peripheral blood-derived osteoclasts contained significantly more nuclei (**d**). 12D and 14D: 12 and 14 days of culture, respectively [143]. Reprinted from Heliyon, Vol 4, Elina Kylmäoja et al. Peripheral blood monocytes show increased osteoclast differentiation potential compared with bone marrow monocytes, Copyright (2018) with permission from Elsevier

Osteoclast differentiation of both HSCs and monocytes derived from bone marrow and peripheral blood has been shown in several in vitro studies [67, 152, 153]. Variations in donor age, health condition, and osteoclastogenesis protocols have resulted in mixed outcomes regarding osteoclast differentiation capacity [143, 166, 167]**.** For instance, a mixture of growth factors such as RANKL, M-CSF, transforming growth factor beta (TGF-β), and dexamethasone has led to the generation of multinuclear cells with higher number of nuclei and an increased expression of osteoclast-specific genes such as tartrate-resistant acid phosphatase (TRACP) 5a and 5b in peripheral blood-derived monocytes compared with bone marrow-derived monocytes (Fig. 3) [143••]. However, no significant differences were reported in bone resorption activity between the used cell types and growth factor combinations [143••]. This study highlighted the importance of carefully considering the combination of chosen growth factors for the osteoclastogenesis of osteoclast precursor cells.

## Conclusion

In vitro bone models provide a platform to study bone physiology including bone remodeling, bone-related diseases, and potential treatments. These models require all three types of cells in bone, namely osteoblasts, osteocytes, and osteoclasts, ideally from individuals to account for donor-specific differences and disease-related cell reactions. To achieve that, it is required to collect progenitor cells from one patient and ideally from one cell source for patient convenience. MSCs and HSCs are progenitor cells of osteoblasts/osteocytes and osteoclasts, respectively, and can be extracted from both bone marrow and peripheral blood as reviewed here. Limited studies directly comparing bone marrow-derived and peripheral blood-derived MSCs and HSCs have shown no significant differences between osteogenesis and osteoclastogenesis of the progenitor cells from both sources. However, many parameters such as medium composition have been reported to affect cell proliferation and their differentiation potential which could make the peripheral blood-derived stem cells superior to the ones from bone marrow. Thus, as both sources have their advantages and disadvantages (Table 2), yet, peripheral blood could be considered as a suitable source for both osteoblast/osteocyte and osteoclast progenitor cells, being less invasive for the patient. In this case, more investigations are needed focusing on extraction and differentiation of both cell types from the same sample of peripheral blood.

**Table 2:** Comparison between MSCs and HSCs/monocytes derived from bone marrow and peripheral blood for in vitro studies

Cell	Source	Collection invasiveness	Frequency of cells	Proliferation capacity	Differentiation potential^a^
Mesenchymal stem cells (MSCs)	Bone marrow	+++	++	+++	+++
	Peripheral blood	+	+	+++	+++
Hematopoietic stem cells (HSCs)/monocytes	Bone marrow	+++	+	+	+++
	Peripheral blood	+	+++	+	+++

^a^The differentiation potential of MSCs and HSCs/monocytes strongly depends on the culture condition

## Data Availability

Not applicable.
